# Accuracy of Intraocular Lens Power Formulas Involving 148 Eyes with Long Axial Lengths: A Retrospective Chart-Review Study

**DOI:** 10.1155/2015/976847

**Published:** 2015-12-17

**Authors:** Chong Chen, Xian Xu, Yuyu Miao, Gaoxin Zheng, Yong Sun, Xun Xu

**Affiliations:** Department of Ophthalmology, Shanghai Key Laboratory of Fundus Disease, Shanghai General Hospital Affiliated to Shanghai Jiao Tong University, Shanghai 200080, China

## Abstract

*Purpose*. This study aims to compare the accuracy of intraocular lens power calculation formulas in eyes with long axial lengths from Chinese patients subjected to cataract surgery.* Methods*. A total of 148 eyes with an axial length of >26 mm from 148 patients who underwent phacoemulsification with intraocular lens implantation were included. The Haigis, Hoffer Q, Holladay 1, and SRK/T formulas were used to calculate the refractive power of the intraocular lenses and the postoperative estimated power.* Results*. Overall, the Haigis formula achieved the lowest level of median absolute error 1.025 D (*P* < 0.01 for Haigis versus each of the other formulas), followed by SRK/T formula (1.040 D). All formulas were least accurate when eyes were with axial length of >33 mm, and median absolute errors were significantly higher for those eyes than eyes with axial length = 26.01–30.00 mm. Absolute error was correlated with axial length for the SRK/T (*r* = 0.212, *P* = 0.010) and Hoffer Q (*r* = 0.223, *P* = 0.007) formulas. For axial lengths > 33 mm, eyes exhibited a postoperative hyperopic refractive error.* Conclusions*. The Haigis and SRK/T formulas may be more suitable for calculating intraocular lens power for eyes with axial lengths ranging from 26 to 33 mm. And for axial length over 33 mm, the Haigis formula could be more accurate.

## 1. Introduction

Myopia is a worldwide health concern, especially in East Asia [1, 2]. In urban areas of Asia, such as Singapore, China, Japan, and Korea, 80–90% of children who complete high school are myopic, and 10–20% have high myopia [3]. Similar trends are seen throughout the world, although they are generally less dramatic. In the United States, the prevalence of myopia, high myopia (−5.01 to −10.00 diopters [D]), and extremely high myopia (more than −10.00 D) are 46.4%, 3.2%, and 0.2%, respectively. Extremely high myopia is also very rare in Europe (1.2%) and Australia (0.3%) [4, 5].

Myopia is commonly defined as spherical equivalent (SE) more than −0.5 D, whereas the definition of high myopia is variable, with a cutoff range of −5.0 to −10.0 D [1]. Some authors [6] define extremely high myopia as an axial length (AL) of >27 mm and a refractive power of more than −10.0 D. However, other authors [7, 8] define extremely high myopia as requiring the implantation of negative-power intraocular lenses (IOLs).

With advances in medical techniques, cataract surgeries are now refractive surgeries rather than rehabilitation surgeries. Thus, accurate IOL power calculations have become extremely important. It is generally accepted that most modern theoretical IOL formulas perform well for eyes of average axial myopia (22.0–24.5 mm) [9]. In cases of high or extremely high axial myopia, the postoperative refractive error may be greater because of difficulties in measuring the AL (the posterior staphyloma makes biometry more difficult) and problems associated with current IOL formulas [5].

In this study, we examined the postoperative refractive status of Chinese patients with an AL of >26 mm after phacoemulsification and IOL implantation, paying particular attention to patients with an AL of >33 mm. We compared the accuracy of several commonly used IOL formulas for predicting postoperative refractive error for individuals with high or extremely high axial myopia. Furthermore, we assessed correlations between AL and postoperative absolute error, which is the absolute value of the postoperative SE minus the predicted postoperative SE.

## 2. Methods

### 2.1. Study Design

This was a retrospective chart-review study. Data were obtained from patient charts and the IOLMaster-500 (Carl Zeiss Meditec, Jena, Germany) at the Shanghai General Hospital Affiliated to Shanghai Jiao Tong University (the National Key Discipline, Shanghai Medical Center for Vision Rehabilitation, Shanghai Eye Institute, Shanghai Key Laboratory of Ocular Fundus Diseases). The IOLMaster uses partial coherence interferometry technology for AL measurements; besides, the automated keratometry (*K*) and anterior chamber depth (ACD, corneal epithelium to lens) measurements allow rapid, noncontact, and accurate measurements of all the required parameters for IOL power calculation [10, 11].

Patient information was anonymized and deidentified prior to analysis. The study was approved by the Ethics Committee of the Shanghai General Hospital Affiliated to Shanghai Jiao Tong University and was conducted in accordance with the Declaration of Helsinki of the World Medical Association.

### 2.2. Participants

Medical records of patients who had undergone phacoemulsification and IOL implantation were reviewed. Patients with an AL of >26 mm (measured using the IOLMaster) were included in the study. In order to avoid duplication/compounding of data with bilateral eyes, only one eye from each study subject was included [12].

Patients were excluded if they had a previous ocular surgery, an eventful cataract surgery, keratoconus, endothelial dystrophy, uveitis, or glaucoma, were with grade IV or above cataract (Lens Opacities Classification System III, LOCS III), or were unable to measure the AL by IOLMaster.

### 2.3. Surgical Procedures

All surgeries were performed by one surgeon (Dr. YS) in the Ophthalmology Department of the General Hospital Affiliated to Shanghai Jiao Tong University. All surgical procedures were conducted under local infiltration anesthesia. A 3 mm wide incision was created in the superior corneal limbus. Phacoemulsification and IOL implantation were performed with continuous curvilinear capsulorhexis. The IOLs were Alcon Acrysofs (MA60MA/SA60AT, Alcon Laboratories, Ft. Worth, TX, USA) or AMO Sensars (AR40E/AR40e/AR40m, Abbott Medical Optics, Irvine, CA USA) with their corresponding optimization constants derived from the manufacturer. The lenses were implanted into the capsular bag.

### 2.4. Main Outcome Measures

An IOLMaster was used to measure the corneal curvature, ACD and AL. Four formulas were used to calculate the refractive power of the IOLs, as well as the estimated postoperative refraction of the eyes by IOLMaster, namely the Haigis [13], Hoffer Q [14], Holladay 1 [15], and SRK/T [16] formulas.

The main assessed parameters were AL, *K*, refractive error (a negative difference implied that the postoperative refractive status was myopic, whereas a positive difference indicated hyperopia), median absolute error (MedAE), and the percentage of eyes with absolute errors within 0.5, 1.0, 2.0, or 3.0 D. Eyes were divided into four groups based on AL, which are across a range of 2.0 mm or 3.0 mm: Group A: AL = 26.01–28.00 mm, Group B: AL = 28.01–30.00 mm, Group C: AL = 30.01–33.00 mm, and Group D: AL = 33.01–36.00 mm.

### 2.5. Statistical Analyses

All statistical analyses were performed using SPSS software, version 22.0 (SPSS, Chicago, USA). Values were recorded as mean ± standard deviation (SD) or median (when data are not a Gaussian distribution) and a 95% confidence interval. A repeated-measures analysis of variance (ANOVA) was performed to assess the overall difference in absolute error among the four formulas and the effect of AL on absolute error in subgroup analyses. The Chi-square test was performed to assess differences between the percentages of eyes with absolute errors of different diopters. Post hoc tests adjusting for multiple comparisons were performed for pairwise comparisons between two formulas. The one-way ANOVA was performed to assess between-group differences in age and corneal power. Correlation between AL and absolute error was evaluated using linear regression analysis. *P* values < 0.05 were considered statistically significant.

## 3. Results

### 3.1. Study Population

A total of 148 eyes from 148 patients who were examined consecutively andmet the inclusion criteria were analyzed. All had undergone phacoemulsification and IOL implantation surgeries. Of the 148 patients, 78 (52.7%) were female. Patient ages ranged from 40 to 88 years (mean = 66.16 ± 10.37 years). The cataract grade ranged from I to III (LOCS III). In general, the study population had a broad range of anatomical variability, with ALs from 26.01 to 35.93 mm (mean = 29.03 ± 2.05 mm) and preoperative corneal curvatures from 39.94 to 47.88 D (mean = 44.15 ± 1.70 D).

For subgroup analyses, eyes were divided into four groups based on AL. There were 57 eyes in Group A, 48 eyes in Group B, 37 eyes in Group C, and 6 eyes in Group D. There were significant differences in age among the four groups (*P* = 0.017; one-way ANOVA). Patients in Group D were significantly younger than patients in Groups A and B (*P* = 0.015 and 0.029, resp.). Besides, there were no significant differences in corneal curvatures among groups (*P* = 0.195; one-way ANOVA). All eyes in Group D had significant posterior scleral staphyloma. Baseline characteristics of the study are summarized in Table 1.

### 3.2. Comparison of Formula Accuracy

**Figure 1:**
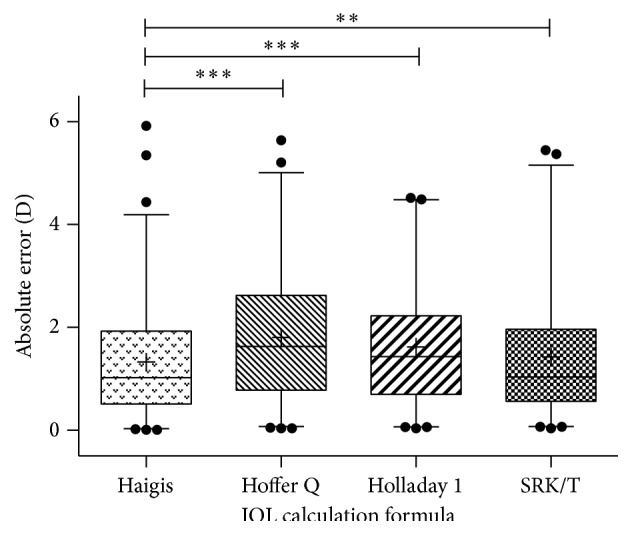
Comparisons of absolute errors in all eyes (*n* = 148). Absolute errors for the four intraocular lens (IOL) calculation formulas. The horizontal lines below and above the main box (whiskers) for each formula represent 2.5 and 97.5 percentile. The symbol + indicates mean absolute error, *∗∗* indicates *P* < 0.01, and *∗∗∗* indicates *P* < 0.001, as determined by a repeated-measures ANOVA test. D, diopters.

For the main outcome of MedAE, the Haigis formula achieved the lowest error of 1.025 D (95% confidence interval = 1.297–1.816 D; *P* < 0.01 for Haigis versus each of the other formulas, Table 2 and Figure 1). In addition, for eyes with absolute errors within 0.5 D of the target, all formulas performed similarly (around 20%), whereas 49.32% and 47.97% of eyes were within 1.0 D of the target using the Haigis and SRK/T formula—42% ~ 52% more than the Hoffer Q formula (33.78%) and Holladay 1 formula (32.43%) (*P* = 0.007 and 0.003 for Haigis versus Hoffer Q and Holladay 1, resp.). These results were essentially consistent across all endpoints for 2.0-D and 3.0-D postoperative refractive thresholds (Table 3).

**Figure 2:**
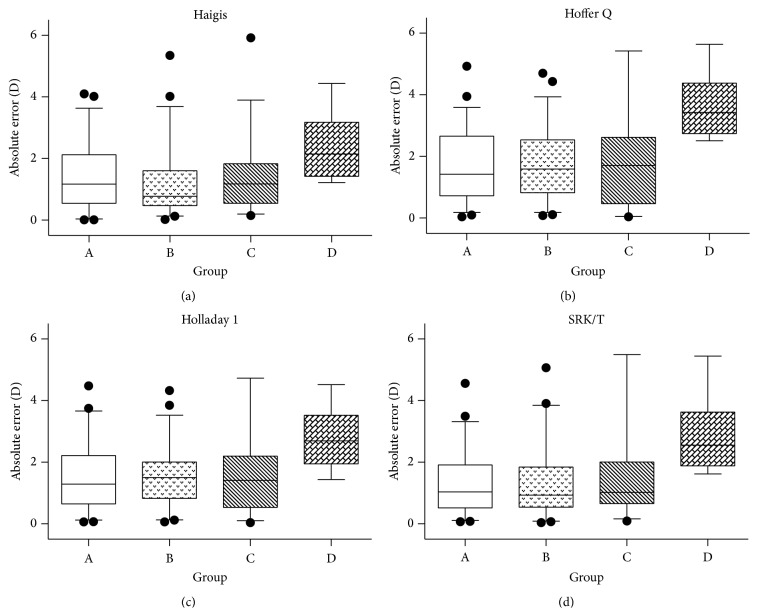
Subgroup analysis: absolute errors for Groups A–D calculated using the four IOL formulas. All four formulas were least accurate for eyes within Group D. The overall median absolute error was significantly higher for Group D than for Groups A, B, and C (*P* = 0.002, 0.002 and 0.010, resp., as determined by a repeated-measures ANOVA test). The whiskers indicate 5 and 95 percentile of absolute errors in each group. D, diopters.

All four formulas were least accurate for eyes within Group D (Figure 2 and Table 4). The MedAE was significantly higher for Group D than for Groups A, B, and C (*P* = 0.002, 0.002, and 0.010, resp.; Table 4). Additionally, for Groups A, B, and C, Haigis and SRK/T formulas were more accurate in calculating the IOL power than the other two formulas (*P* < 0.05, repeated-measures ANOVA; Table 4), while there were no significant differences between Haigis and SRK/T formulas (*P* > 0.05). However, for Group D, Haigis was more accurate than each of the other formula (*P* < 0.01). Three months after the operation, all the six eyes in Group D were hyperopic, for which the IOL power was calculated using the four formulas.

### 3.3. Correlation between AL and Absolute Error

**Figure 3:**
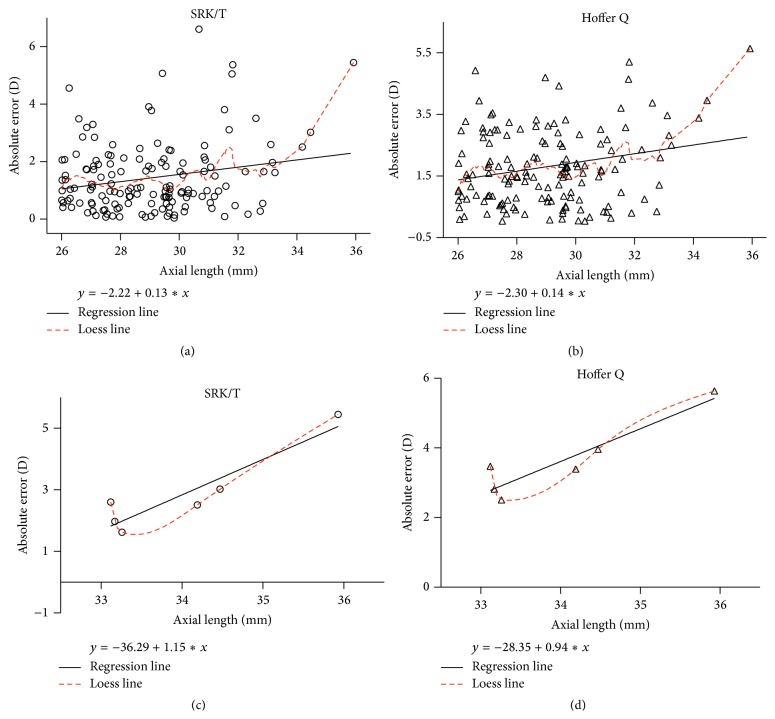
Correlations between axial length and absolute error. The associations between axial length and absolute error were analyzed using absolute errors derived from (a) the SRK/T formula (*r* = 0.212; *P* = 0.010; regression equation: *y* = −2.22 + 0.13*x*) and (b) the Hoffer Q formula (*r* = 0.223; *P* = 0.007; regression equation: *y* = −2.30 + 0.14*x*). Similar analyses were carried out with Group D data only using (c) the SRK/T formula (*r* = 0.926; *P* = 0.008; regression equation: *y* = −36.29 + 1.15*x*) and (d) the Hoffer Q formula (*r* = 0.928; *P* = 0.008; regression equation: *y* = −28.35 + 0.94*x*).

To determine whether AL correlates with postoperative refractive outcome, the correlation between AL and absolute error was evaluated using linear regression analysis. Absolute error was associated with AL when the Hoffer Q or SRK/T formulas were used (SRK/T: Pearson correlation *r* = 0.212, *P* = 0.010; Hoffer Q: *r* = 0.223, *P* = 0.007; Figures 3(a) and 3(b)). None of the other formulas revealed significant associations between AL and absolute error (Holladay 1: *r* = 0.150 and *P* = 0.070; Haigis: *r* = 0.106 and *P* = 0.198). When we focused on data from Group D, much stronger associations between AL and absolute error were found (SRK/T: *r* = 0.926 and *P* = 0.008; Hoffer Q: *r* = 0.928 and *P* = 0.008; Figures 3(c) and 3(d)).

## 4. Discussion

Refractive status is a complex variable determined by the optical power of the cornea and the lens and the AL of the eye (with its component parts ACD, lens thickness, and vitreous chamber depth) [3]. Although there is no clear definition of extremely high axial myopia, it is well established that the higher the refractive power and the longer the AL are, the more significant the deviation in AL measurement and the refractive power of the IOL calculation will be [17, 18].

The widespread application of phacoemulsification and IOL implantation cataract surgery has led to improved surgical techniques and fewer surgical complications. Thus, the postoperative refractive status is less affected by surgical factors than it has been in the past. Accuracy of the IOL power calculation is now the most important factor affecting the postoperative refractive status. Moreover, the choice of IOL formula is closely related to the accuracy of IOL power calculation.

In 1990, Sanders et al. [19] reported that the SRK/T formula is marginally better for eyes with high axial myopia. That study, however, included very few eyes with an AL of ≥28.4 mm, because among these patients from Europe and America the proportion of eyes with an AL of >27 mm or >28.4 mm was only 1.0% or 0.1%, respectively. However, China has a very high percentage of people with a long AL and extremely high axial myopia [3].

During the past decade, relationships between eyes with a long AL and postoperative refractive error have been examined in a range of ethnicities [18, 20–25]. These analyses did not, however, produce a consensus concerning the most accurate formula in predicting postoperative refractive error in long eyes. Importantly, the eyes evaluated rarely had an AL of >30 mm.

Our study found that, overall, the Haigis formula resulted in the lowest MedAE (1.025 D) in high and extremely high myopic Chinese eyes with an AL of >26 mm (mean AL = 29.02 mm). The SRK/T formula generated the second most accurate results (1.040 D), whereas the Hoffer Q was the least accurate in all subgroups. The Haigis formula is a fourth-generation formula and may have performed better in highly myopic eyes because it uses three constants, *a*_0_, *a*_1_, and *a*_2_, to predict the effective lens position (ELP), where ELP = *a*_0_ + (*a*_1_ × ACD_preoperative_)+(*a*_2_ × AL). Third-generation formulas, such as Hoffer Q, Holladay 1, and SRK/T, rely on AL and central corneal power to calculate the ELP without actually measuring the ACD. This approach may be less accurate for short or long eyes [13, 18].

We further illustrated that longer ocular ALs are associated with less-accurate predictions of postoperative refractive status, especially for eyes with an AL of >33 mm. We draw this conclusion from the regression equations shown in Figure 3. For the SRK/T and Hoffer Q formulas, a 1 mm increase in AL resulted in an absolute error increase of ~0.1 D (for AL > 26 mm). For eyes with an AL of >33 mm, however, a 1 mm increase in AL resulted in an absolute error increase of 1.15 D for SRK/T and 0.94 D for Hoffer Q.

In addition, our results confirmed earlier findings that the implantation of low-power IOLs (including negatively powered IOLs) into highly myopic eyes resulted in hyperopic refractive errors [17, 18, 20, 26–29]. We found that most eyes with an AL of >33 mm presented with postoperative hyperopia of +2.0 D to +3.0 D. Haigis [5, 17] indicated that plus-IOLs and minus-IOLs should be characterized by different sets of IOL constants. It has recently been demonstrated that AL-dependent hyperopic refractive errors are primarily caused by the use of positive-power IOL constants for both positive-power and negative-power IOLs [17].

We recommend that, for eyes with extremely long ALs, preservation of postoperative myopia of −2.0 to −3.0 D (or more) should be a preoperative consideration when calculating the refractive power of the implanted IOLs. This is consistent with the near-sighted lifestyle of patients and can reduce the possibility of postoperative hyperopia resulting from errors associated with AL measurement and current IOL formulas. Furthermore, extremely long ALs are often associated with fundus lesions, which reduce distance vision. As distance vision for these patients is probably critically reduced, trying to improve postoperative near vision may represent a good option.

## 5. Conclusions

Our findings suggest that the Haigis and SRK/T formulas perform better for calculating the IOL power for Chinese patients whose eyes have an AL ranging within 26~33 mm (MedAEs ~ 1.0 D; Table 4). Therefore, to achieve a target refraction of −3.00 D in Chinese eyes with an AL of 26.01–33.00 mm, we suggest setting a postoperative target around −4.00 D, using Haigis or SRK/T formulas. In addition, the Haigis may be the best formula for eyes with an AL of >33 mm; however, the postoperative absolute error increased to ~2.0 D even when using the Haigis formula (Table 4). Hence, for eyes with an AL of 33.00–36.00 mm, we recommend setting a postoperative target around −5.00 D using Haigis formula. Selecting higher IOL powers is often preferred, leaving the patient slightly myopic rather than hyperopic.

Further studies using other IOL calculating formulas should be conducted to compare the accuracy for extremely high axial myopia [10], and predictive models should be improved to increase the accuracy of IOL calculations.

## Figures and Tables

**Table 1:** Baseline characteristics of eyes included in the study.

AL (mm)	Eyes (number)	Patient age (yr)	Mean AL (mm)	Mean *K* (D)
Group A				
26.01–27.00	24	68.92 ± 10.15	26.39 ± 0.33	44.28 ± 1.92
27.01–28.00	33	67.67 ± 9.24	27.46 ± 0.32	44.06 ± 1.97
Subtotal	**57**	**68.19 ± 9.56**	**27.01 ± 0.63**	**44.15 ± 1.94**
*P* value^†^		**0.015** ^*∗*^	/	**0.137**
Group B				
28.01–29.00	17	68.29 ± 11.85	28.54 ± 0.29	44.61 ± 1.84
29.01–30.00	31	66.58 ± 10.45	29.49 ± 0.24	44.39 ± 1.32
Subtotal	**48**	**67.19 ± 10.87**	**29.16 ± 0.53**	**44.47 ± 1.51**
*P* value^†^		**0.029** ^*∗*^	/	**0.057**
Group C				
30.01–31.00	18	63.50 ± 10.55	30.45 ± 0.34	43.67 ± 1.70
31.01–32.00	13	64.92 ± 10.89	31.45 ± 0.30	44.17 ± 1.73
32.01–33.00	6	58.00 ± 9.83	32.61 ± 0.26	44.22 ± 0.86
Subtotal	**37**	**63.11 ± 10.47**	**31.15 ± 0.86**	**43.93 ± 1.59**
*P* value^†^		**0.210**	/	**0.244**
Group D				
33.01–36.00	6	57.50 ± 4.68	34.02 ± 1.09	43.07 ± 0.58
Subtotal	**6**	**57.50 ± 4.68**	**34.02 ± 1.09**	**43.07 ± 0.58**

Total	**148**	**66.16 ± 10.37**	**29.03 ± 2.05**	**44.15 ± 1.70**
*P* value^‡^		**0.017** ^*∗*^	/	**0.195**

AL, axial length; *K*, keratometric reading; D, diopters.

^†^Compared with Group D (one-way ANOVA).

^‡^Compared among the four groups (one-way ANOVA).

*∗* indicates *P* < 0.05.

**Table 2:** Absolute error (D) for each formula.

Formula	Median absolute error^†^	95% CI	Range	*P* value^‡^
Haigis	1.025	1.297–1.816	0.01–5.92	/
Hoffer Q	1.635	1.925–2.042	0.04–7.37	<0.001^*∗∗∗*^
Holladay 1	1.435	1.610–2.149	0.04–6.87	<0.001^*∗∗∗*^
SRK/T	1.040	1.479–2.042	0.04–6.61	0.002^*∗∗*^

^†^Absolute error = actual postoperative spherical equivalent − predicted spherical equivalent.

^‡^Compared with results achieved using the Haigis formula (repeated-measures ANOVA).

D, diopters; CI, confidence interval.

*∗∗* indicates *P* < 0.01; *∗∗∗* indicates *P* < 0.001.

**Table 3:** Percentages of eyes with different absolute errors at different refractive thresholds.

Formula	Percentages of eyes with indicated absolute error (*P* value^†^)			
	<0.5 D	<1.0 D	<2.0 D	<3.0 D
Haigis	23.65%	49.32%	77.03%	91.89%
Hoffer Q	16.89% (0.148)	33.78% (0.007)	63.51% (0.011)	81.76% (0.010)
Holladay 1	18.24% (0.253)	32.43% (0.003)	68.24% (0.090)	87.16% (0.184)
SRK/T	19.60% (0.397)	47.97% (0.816)	76.35% (0.891)	89.86% (0.545)

^†^Compared with results achieved using the Haigis formula (Chi-square test). D, diopters.

**Table 4:** Median absolute errors (D) calculated by each formula for Groups A–D.

Formula	Group A (*n* = 57)	Group B (*n* = 48)	Group C (*n* = 37)	Group D (*n* = 6)
Haigis	1.080	0.805	1.160	2.145
Hoffer Q	1.420	1.635	1.710	3.430
Holladay	1.290	1.530	1.410	2.695
SRK/T	1.040	0.975	0.990	2.555
*P*-value^†^	0.002	0.002	0.010	/

^†^Compared with the results achieved with Group D (repeated-measures ANOVA).

Group A: AL = 26.01–28.00 mm, Group B: AL = 28.01–30.00 mm, Group C: AL = 30.01–33.00 mm, and Group D: AL = 33.01–36.00 mm.

D, diopters.
